# RhoA/ROCK Regulates Prion Pathogenesis by Controlling Connexin 43 Activity

**DOI:** 10.3390/ijms21041255

**Published:** 2020-02-13

**Authors:** Hee-Jun Kim, Mo-Jong Kim, Mohd Najib Mostafa, Jeong-Ho Park, Hong-Seok Choi, Yong-Sun Kim, Eun-Kyoung Choi

**Affiliations:** 1 Ilsong Institute of Life Science, Hallym University, Anyang, Gyeonggi-do 14066, Korea; hijuni@hallym.ac.kr (H.-J.K.); hanbami0730@hallym.ac.kr (M.-J.K.); mnajib94@gmail.com (M.N.M.); jh0622@naver.com (J.-H.P.); choihs@hallym.ac.kr (H.-S.C.); yskim@hallym.ac.kr (Y.-S.K.); 2 Department of Biomedical Gerontology, Graduate School of Hallym University, Chuncheon, Gangwon-do 24252, Korea; 3 Department of Microbiology, College of Medicine, Hallym University, Chuncheon, Gangwon-do 24252, Korea

**Keywords:** prion diseases, RhoA, ROCK, Cx43, p190RhoGAP, neuronal cell death

## Abstract

Scrapie infection, which converts cellular prion protein (PrP^C^) into the pathological and infectious isoform (PrP^Sc^), leads to neuronal cell death, glial cell activation and PrP^Sc^ accumulation. Previous studies reported that PrP^C^ regulates RhoA/Rho-associated kinase (ROCK) signaling and that connexin 43 (Cx43) expression is upregulated in in vitro and in vivo prion-infected models. However, whether there is a link between RhoA/ROCK and Cx43 in prion disease pathogenesis is uncertain. Here, we investigated the role of RhoA/ROCK signaling and Cx43 in prion diseases using in vitro and in vivo models. Scrapie infection induced RhoA activation, accompanied by increased phosphorylation of LIM kinase 1/2 (LIMK1/2) at Thr508/Thr505 and cofilin at Ser3 and reduced phosphorylation of RhoA at Ser188 in hippocampal neuronal cells and brains of mice. Scrapie infection-induced RhoA activation also resulted in PrP^Sc^ accumulation followed by a reduction in the interaction between RhoA and p190RhoGAP (a GTPase-activating protein). Interestingly, scrapie infection significantly enhanced the interaction between RhoA and Cx43. Moreover, RhoA and Cx43 colocalization was more visible in both the membrane and cytoplasm of scrapie-infected hippocampal neuronal cells than in controls. Finally, RhoA and ROCK inhibition reduced PrP^Sc^ accumulation and the RhoA/Cx43 interaction, leading to decreased Cx43 hemichannel activity in scrapie-infected hippocampal neuronal cells. These findings suggest that RhoA/ROCK regulates Cx43 activity, which may have an important role in the pathogenesis of prion disease.

## 1. Introduction

Prion disease, a form of transmissible spongiform encephalopathy, is characterized by progressive neuronal degeneration caused by conformational changes in the cellular prion protein (PrP^C^) into a misfolded and aggregated form (PrP^Sc^), which accumulates in the brain [1,2,3,4]. The pathological symptoms of prion disease are distinguished by its transmissibility, characteristic spongiform changes associated with synapse dysfunction, axon retraction and loss of neuronal polarity preceding neuronal cell loss, astrocytosis and amyloid plaque formation, and the prolonged incubation period between exposure and symptom onset. These changes lead to the release of inflammatory molecules, including proinflammatory cytokines, reactive oxygen species, proteases, and complement proteins that induce neuronal damage and removal of damaged cells [5,6].

PrP^C^ has been reported to contribute to neuronal differentiation in various neuronal cell lines by promoting neurite sprouting, extension, stability, and plasticity of neuronal polarity [7], which may depend on interactions with various proteins, including neural cell adhesion molecules (NCAMs) [8,9], heparan sulfate proteoglycans (HSPGs) [10,11], stress-inducible protein-1 (STI1) [12], growth factor receptor-bound protein 2 (Grb2) [13], caveolin [14], extracellular matrix (ECM) proteins [15,16], and RhoA [17,18]. In particular, PrP^C^ regulates RhoA activity and the RhoA-Rho-associated kinase (ROCK)-LIM kinase (LIMK)-cofilin pathway by facilitating the interaction between RhoA and p190RhoGAP in vitro and in vivo [17,18]. Scrapie infection alters neuronal differentiation and the actin network by overactivating the RhoA/ROCK and RhoA-ROCK-LIMK-cofilin signaling pathways. Moreover, ROCK activation and ROCK-3-phosphoinositide-dependent kinase 1 (PDK1) complex formation contribute to the regulation of neuronal polarity and the generation of pathogenic prions [6].

The RhoA/ROCK signaling pathway has been associated with diverse cellular functions, including actin cytoskeleton reorganization, motility, morphology, polarity, cell division, cell cycle progression, and membrane trafficking [19,20,21]. In the central nervous system (CNS), RhoA/ROCK and its regulatory protein, p190RhoGAP play an important role in neuronal differentiation, neurite outgrowth, neuronal migration, axon growth and dendritic spine formation and maintenance [22].

Gap junction alpha-1 protein (GJA1), known as Cx43, forms intercellular channels that connect adjacent cells to permit the exchange of low molecular weight molecules, such as adenosine triphosphate, glutamate, and Ca^2+^ ions, to maintain homeostasis [23,24,25]. Cx43 is widely expressed in the CNS, especially neurons, microglia, astrocytes, and oligodendrocytes [26,27,28], and has been shown to be involved in various neurodegenerative diseases, including Alzheimer’s disease [29], Parkinson’s disease [30], multiple sclerosis [31], epilepsy [32] and prion diseases [33]. In addition, a report demonstrated that Cx43 channel permeability is controlled by RhoA/ROCK activity and F-actin formation [34]. Furthermore, an immunoprecipitation-based proteomics approach in samples from healthy and osteoarthritis patients revealed that Cx43 interacts with RhoA [35]. However, the regulatory roles of Cx43- and RhoA/ROCK-related signaling molecules in prion diseases remain unknown. Therefore, the present study aimed to determine the regulatory role of the RhoA/ROCK and Cx43 signaling pathways in prion pathogenesis in both in vitro and in vivo models of prion disease.

## 2. Results

### 2.1. Scrapie Infection Alters RhoA Activity and the RhoA-ROCK-LIMK-Cofilin Pathway

**Figure 1:**
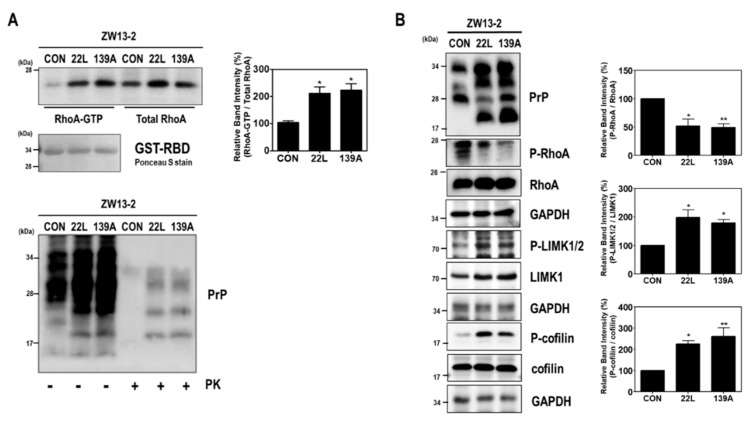
Scrapie infection altered RhoA activity and the RhoA-ROCK-LIMK-cofilin pathway in hippocampal neuronal cell lines. (**A**) Detection of RhoA-GTP by glutathione S-transferase (GST)-rhotekin-rho-binding domain (RBD) pull-down assay in ZW13-2 hippocampal neuronal cell lines (control, CON) with or without 22L or 139A scrapie infection. The level of RhoA-GTP was determined by Western blot with the anti-RhoA antibody after a pull-down assay (upper left panel). For PrP^Sc^ detection, cell lysates (40 μg of total protein) were digested with proteinase K (PK; 20 μg/mL) for 30 min at 37 °C and detected using anti-PrP (3F10) antibody (bottom panel). (**B**) Phosphorylation levels of RhoA, LIMK1/2, and cofilin in ZW13-2 cells with or without 22L or 139A scrapie infection were analyzed by Western blot. The intensities of the bands in each panel were measured and quantified for each group, and the values are expressed as the mean ± SEM of three independent experiments. Statistical differences were determined by one-way ANOVA with Tukey’s post hoc test (*n* = 3, * *p* < 0.05; ** *p* < 0.01).

**Figure 2:**
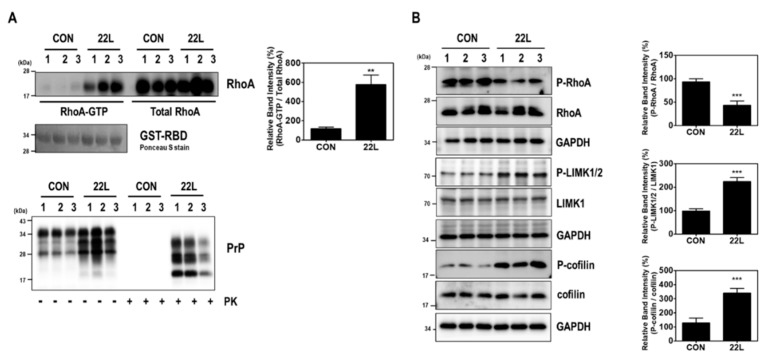
Scrapie infection induced RhoA activation and the RhoA-ROCK-LIMK-cofilin pathway in the brains of scrapie-infected mice. (**A**) Detection of RhoA-GTP levels in the brains of C57BL/6J (CON) and scrapie-infected (22L) mice (upper left panel). To detect PrP^Sc^, brain homogenates (40 μg of total protein) were digested with PK (20 μg/mL) for 30 min at 37 °C and detected using anti-PrP (3F10) antibody (bottom panel). (**B**) Phosphorylation of RhoA, LIMK, and cofilin was assessed in the whole-brain lysates of CON and 22L mice. The intensities of the bands in each panel were measured and quantified for each group, and the values were expressed as the mean ± SEM of three independent experiments. Statistical differences were determined by one-way ANOVA with Tukey’s post hoc test (*n* = 3 per group, ** *p* < 0.01; *** *p* < 0.001).

We first investigated the effect of scrapie infection on RhoA activity and the RhoA-ROCK-LIMK-cofilin pathway. As shown in Figure 1, compared to levels in control hippocampal neuronal cells, increased RhoA-guanosine triphosphate (GTP) levels were found in scrapie-infected mouse hippocampal neuronal cells (Figure 1A), which were used as an in vitro model of prion replication after infection with either of two mouse-derived scrapie strains (22L or 139A) [33,36]. Moreover, scrapie infection induced the phosphorylation of LIMK1/2 at Thr508 and Thr505 (P-LIMK1/2) and cofilin at Ser3 (P-cofilin) with a reduction in the phosphorylation of RhoA at Ser188 (P-RhoA) (Figure 1B). To confirm these results, we examined the effect of PrP^Sc^ on RhoA activity and the phosphorylation levels of RhoA downstream proteins in the brains of control and 22L scrapie-infected mice. As expected, we observed an increase in RhoA-GTP levels (Figure 2A) as well as a decrease in P-RhoA levels and increases in P-LIMK1/2 and P-cofilin levels (Figure 2B) in the brains of scrapie-infected mice compared to those of control mice. These findings indicate that PrP^Sc^ induces RhoA activation and subsequently affects its downstream regulatory proteins, including LIMK and cofilin, after scrapie infection.

### 2.2. Scrapie Infection Controls F-Actin Formation through RhoA Activity

**Figure 3:**
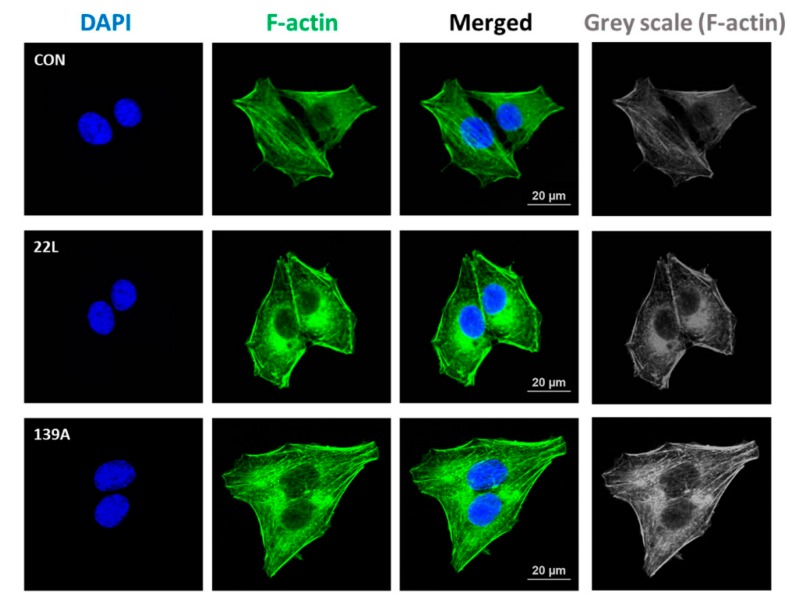
Scrapie infection increased F-actin formation. Immunocytochemical staining for F-actin in ZW13-2 cells with or without 22L or 139A scrapie infection. Cells were fixed with 4% paraformaldehyde (PFA) and permeabilized with 0.2% Triton X-100 in PBS. F-actin was stained with Alexa Fluor 488-phalloidin, and 4′,6-diamidino-2-phenylindole (DAPI) was used to counterstain the nuclei. All pictures are representative of multiple images from three independent experiments (scale bars, 20 μm).

Previous studies have reported that RhoA activation plays a role in the regulation of cytoskeleton reorganization and rearrangement by facilitating the formation of actin stress fibers [21,37]. Thus, we investigated the effect of PrP^Sc^ on the formation of actin stress fibers in hippocampal neuronal cells using fluorescent staining of F-actin (Alexa Fluor 488-conjugated phalloidin). As shown in Figure 3, F-actin formation was more strongly detected in scrapie-infected hippocampal neuronal cells than in control cells. Furthermore, we examined whether RhoA-mediated signaling is responsible for F-actin formation in control and 22L scrapie-infected cells. Cells were pretreated with Y27632 (an inhibitor of ROCK) and Tat-C3 (a specific inhibitor of RhoA, ADP-ribosylation at Asn41). Interestingly, RhoA and ROCK inhibition decreased F-actin formation in scrapie-infected hippocampal neuronal cells (Figure S1). These findings indicate that PrP^Sc^ is involved in F-actin formation by regulating RhoA/ROCK activity.

### 2.3. Scrapie Infection Induces the Activation of RhoA and the RhoA-ROCK-LIMK-Cofilin Pathway by Reducing the Interaction between RhoA and p190RhoGAP

**Figure 4:**
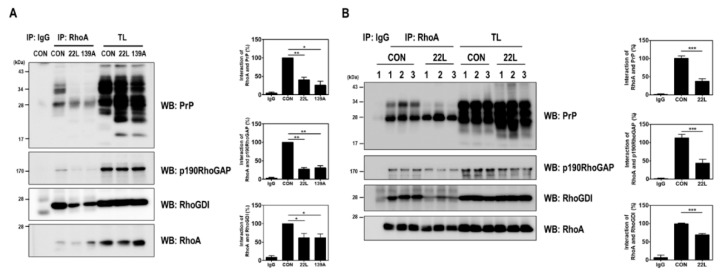
Scrapie infection impaired the RhoA-PrP and RhoA-RhoA effector protein interactions. (**A** and **B**) Coimmunoprecipitation of RhoA with PrP and RhoA-regulated proteins in ZW13-2 cells with or without 22L or 139A scrapie infection (**A**) and whole-brain lysates of control (CON) and 22L-infected mice (**B**). IP, immunoprecipitation; TL, total lysates; WB, Western blot analysis. IgG was used as a control IP. The intensities of the bands in each panel were measured and quantified for each group, and the values are expressed as the mean SEM of three independent experiments. Statistical differences were determined by one-way ANOVA with Tukey’s post hoc test (*n* = 3, * *p* < 0.05; ** *p* < 0.01, *** *p* < 0.001).

PrP^C^ plays a pivotal regulatory role in regulating the RhoA/ROCK signaling pathway in neuritogenesis by interacting with proteins, including *β*1-integrin, PDK1, and RhoA, in neuronal cell lines [6,7,17]. In particular, PrP^C^ interacts with RhoA and p190RhoGAP and controls RhoA activity in hippocampal neuronal cells [17]. Therefore, we examined whether scrapie infection affects the interaction between RhoA and p190RhoGAP, which can regulate the RhoA/ROCK signaling pathway, by performing a coimmunoprecipitation assay using scrapie-infected hippocampal neuronal cells and the brains of control and scrapie-infected mice. As shown in Figure 4, the RhoA-p190RhoGAP interaction was significantly reduced in the brains of scrapie-infected mice compared to that in the brains of control mice. Taken together, these findings suggest that scrapie infection efficiently inactivates RhoA, thereby affecting its downstream regulatory proteins, including LIMK and cofilin, and that PrP^Sc^ impairs the interaction between RhoA and p190RhoGAP.

### 2.4. Scrapie Infection Enhances the Interaction between RhoA and Cx43

**Figure 5:**
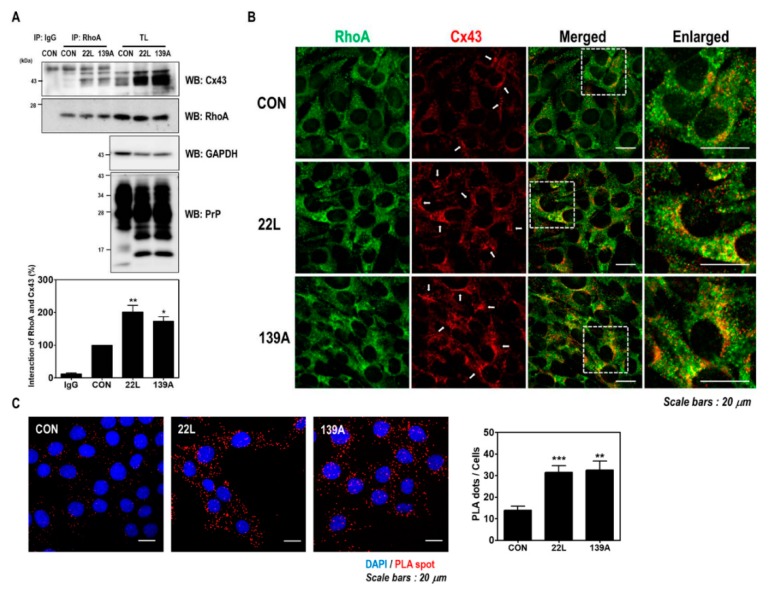
Scrapie infection enhanced the interaction between RhoA and Cx43. (**A**) Coimmunoprecipitation of RhoA with Cx43 in ZW13-2 cells with or without 22L or 139A scrapie infection. IP, immunoprecipitation; TL, total lysates; WB, Western blot analysis. IgG was used as a control IP. The intensities of the bands in each panel were measured and quantified for each group, and the values were expressed as the mean ± SEM of three independent experiments. Statistical differences were determined by one-way ANOVA with Tukey’s post hoc test (*n* = 3, * *p* < 0.05; ** *p* < 0.01). (**B** and **C**) The colocalization of RhoA with Cx43. ZW13-2 cells with or without the 22L scrapie strain was determined by double immunofluorescence staining (**B**) and in situ PLA (**C**) using confocal microscopy. Green, RhoA; red, Cx43. DAPI (blue) was used to counterstain the nuclei. (Scale bars, 20 μm). The interaction was measured by an in situ PLA. Statistical differences were determined by one-way ANOVA with Tukey’s post hoc test (*n* = 3, ** *p* < 0.01, *** *p* < 0.001).

Previous studies have reported that scrapie infection upregulates Cx43 expression through the c-Jun N-terminal kinase (JNK) signaling pathway in the brains of mice and hippocampal neuronal cells [33]. Furthermore, RhoA was previously identified as a Cx43-interacting protein by proteomic analysis [35]. Thus, we investigated whether scrapie infection affects the interaction between RhoA and Cx43 in hippocampal neuronal cells and the brains of mice. As shown in Figure 5A, an increased interaction between RhoA and Cx43 was found after scrapie infection. Furthermore, RhoA and Cx43 colocalization was more intensely detected in the membrane and cytoplasm in scrapie-infected cells than in control cells (Figure 5B). In addition, the PLA, which evaluates the interaction of two proteins in situ, showed a significantly increased interaction between RhoA and Cx43 after scrapie infection (Figure 5C). These results indicate that scrapie infection enhances the interaction between RhoA and Cx43.

### 2.5. Inhibition of RhoA and ROCK Reduces PrP^Sc^ Accumulation and the RhoA-Cx43 Interaction in Scrapie-Infected Hippocampal Neuronal Cells

**Figure 6:**
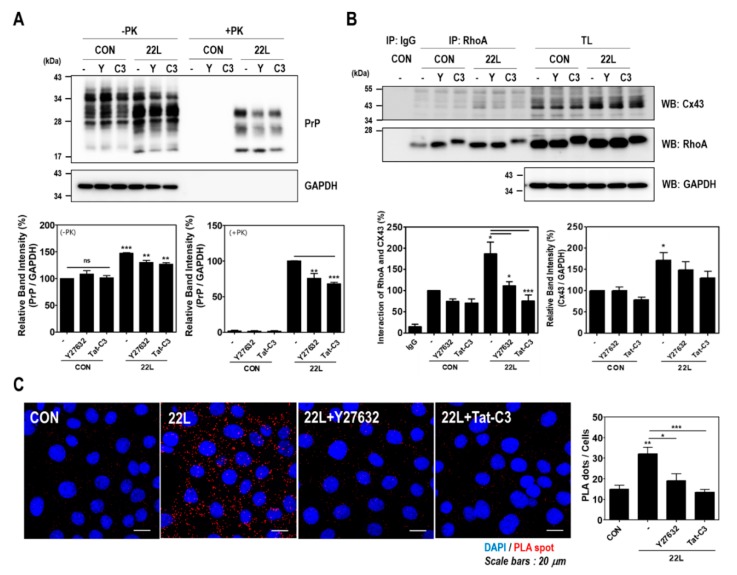
Inhibition of RhoA and ROCK affected PrP^Sc^ accumulation and the RhoA-Cx43 interaction in scrapie-infected ZW13-2 hippocampal neuronal cells. (A and B) Control (CON) and 22L scrapie-infected cells were incubated with or without 10 μM Y27632 and 1 μg/mL Tat-C3 for 24 h. To detect PrP^Sc^ accumulation (**A**), cell lysates were treated with PK (20 μg/mL) for 30 min. Coimmunoprecipitation of RhoA with Cx43 (**B**) was performed using the anti-RhoA antibody and analyzed by Western blot with anti-CX43 and anti-RhoA antibodies. Glyceraldehyde 3-phosphate dehydrogenase (GAPDH) was used as a loading control. The intensities of the bands in each panel were measured and quantified for each group, and the values are expressed as the mean ± SEM of three independent experiments. Statistical data were obtained by one-way ANOVA with Tukey’s post hoc test (*n* = 3, * *p* < 0.05; ** *p* < 0.01; *** *p* < 0.001). (**C**) The interaction between RhoA and Cx43 was assessed in control and 22L scrapie infected cells with or without 10 μM Y27632 and 1 μg/mL Tat-C3. The interaction was measured by an in situ PLA. Statistical differences were determined by one-way ANOVA with Tukey’s post hoc test (*n* = 3, * *p* < 0.05; ** *p* < 0.01, *** *p* < 0.001).

To determine whether RhoA-mediated signaling is responsible for the induction of RhoA activity and the enhancement of the interaction between RhoA and Cx43 after scrapie infection, cells were pretreated with Y27632 and Tat-C3. Interestingly, Y27632 and Tat-C3 treatment significantly decreased PrP^Sc^ accumulation, Cx43 expression, and the interaction between RhoA and Cx43 in scrapie-infected hippocampal neuronal cells (Figure 6A,B). Supporting these results, we found similar results using an in situ PLA (Figure 6C). Furthermore, RhoA and ROCK inhibition reduced JNK phosphorylation in scrapie-infected hippocampal neuronal cells (Figure S2). These data indicate that RhoA/ROCK regulates Cx43 expression and its signaling pathway, which are involved in the pathogenesis of prion disease.

### 2.6. Inhibition of RhoA and ROCK Reduces EtBr Uptake and Dye Transfer in Scrapie-Infected Hippocampal Neuronal Cells

**Figure 7:**
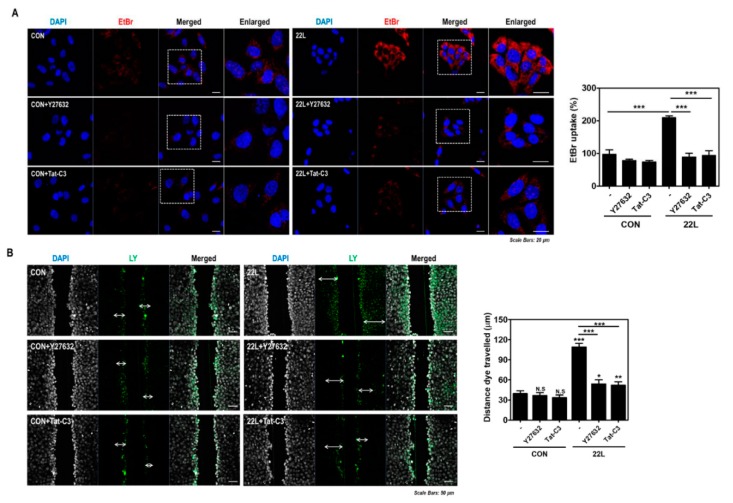
Inhibition of RhoA and ROCK reduced hemichannel function in scrapie-infected cells. (**A**) ZW13-2 cells with or without 22L scrapie infection were pretreated with or without 10 μM Y27632 (middle panels) or 1 μg/mL Tat-C3 (bottom panels) for 6 h and then incubated with 5 μM EtBr for 5 min. The fluorescence signal was analyzed using a confocal microscope. Bar graph illustrates EtBr uptake normalized to and statistically compared to control. (**B**) Cells with or without 22L scrapie infection were incubated with 0.1% Lucifer yellow (LY) with or without Y27632 (10 μM) or Tat-C3 (1 μg/mL) treatment for 6 h. LY fluorescence was analyzed using a confocal microscope. Bar graph illustrates EtBr uptake normalized to and statistically compared to control. Statistical differences were determined by one-way ANOVA with Tukey’s *post hoc* test (*n* = 5, * *p* < 0.05; ** *p* < 0.01; *** *p* < 0.001).

Previous studies have reported that RhoA/ROCK activity is involved in Cx43 hemichannel function and expression in various cell lines, the corneal epithelium and the cardiac conduction system [34,38,39,40,41]. Therefore, we examined whether RhoA/ROCK inhibition modulates Cx43 hemichannel function in scrapie-infected hippocampal neuronal cells and found that RhoA/ROCK inhibition significantly decreased EtBr uptake and dye transfer in cells (Figure 7A,B). These data suggest that the inhibition of RhoA/ROCK signaling results in reduction of Cx43 hemichannel function; consequently, scrapie infection regulates the RhoA/ROCK-Cx43 signaling pathway.

## 3. Discussion

In prion diseases, protease-resistant and infectious PrP^Sc^ induces spongiform encephalopathy with spontaneous neurodegeneration, and disease-associated genetic mutations of PrP^C^ lead to severe ataxia, apoptosis, and extensive central and peripheral myelin degeneration [42,43]. A recent study demonstrated that overexpression of the disease-associated mutants of PrP^C^ (P102L and MΔ8) impaired neuronal differentiation because of the failure to inactivate RhoA/ROCK and reduced the coimmunoprecipitation of RhoA and p190RhoGAP in PC12 cells [17,18]. In addition, scrapie infection upregulated the expression of Cx43 through the JNK signaling pathway in both in vitro and in vivo models of prion disease [33].

**Figure 8:**
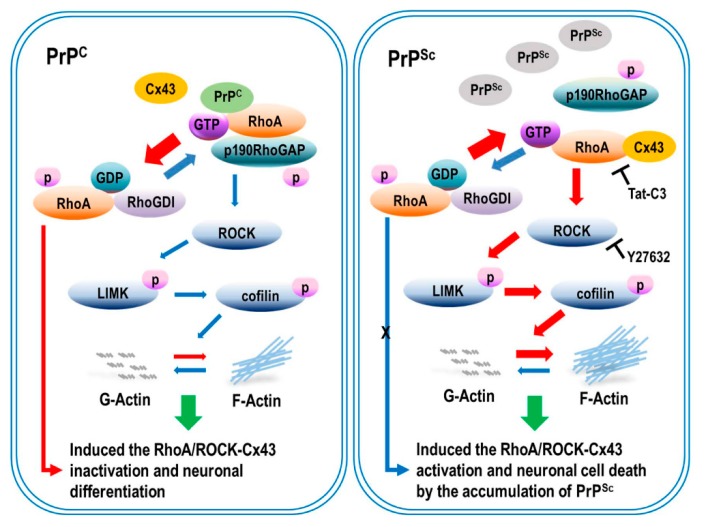
Scrapie infection regulated the activation of RhoA/ROCK-Cx43 signaling and affected PrP^Sc^ accumulation and hemichannel function. Scrapie infection decreased the phosphorylation of RhoA, reducing the interaction between RhoA and p190RhoGAP, leading to the activation of RhoA and its downstream effector proteins. Moreover, scrapie infection increased PrP^Sc^ accumulation and hemichannel function. Subsequently, RhoA/ROCK-Cx43 activation enhanced actin polymerization and decreased neurite outgrowth. However, inhibition of RhoA/ROCK signaling prevented the activation of Cx43 and reduced the interaction between RhoA and Cx43, which prevented RhoA/ROCK activation, PrP^Sc^ accumulation, and hemichannel function by interfering with the interactions.

In this study, we demonstrated a novel mechanism by which scrapie infection induced the activation of the RhoA/ROCK-Cx43 signaling pathway, which controls the accumulation of PrP^Sc^ and the functional properties of hemichannels and has an important role in the pathogenesis of prion diseases (Figure 8). Previous studies have reported p190RhoGAP knockout results in brain morphogenesis defects and that the absence of p190RhoGAPs causes a failure in hemisphere fusion, eventually leading to agenesis and neural tube defects [44,45]. Collectively, these reports suggest that p190RhoGAPs play a significant role in RhoA inactivation, which eventually helps in neural protection. Since neuronal loss is one of the major pathological changes in prion diseases, RhoA activation may participate in prion pathogenesis. Intriguingly, our study demonstrated that scrapie infection significantly increased the level of RhoA-GTP and phosphorylation of LIMK1/2 and cofilin without changing the total levels of RhoA, LIMK1 or cofilin in vitro and in vivo (Figure 1 and Figure 2). Moreover, PrP^C^ regulates RhoA activity by modulating the interaction between RhoA and p190RhoGAP, in concert with increasing phosphorylation of RhoA at Ser 188 and decreasing phosphorylation of downstream effector proteins (i.e., LIMK and cofilin). In addition, the interaction of RhoA and p190RhoGAP is known to regulate neurite outgrowth in PC12 cells through RhoA inactivation [17,46,47]. Similarly, we also demonstrated that scrapie infection decreased the interaction between RhoA and p190RhoGAP (Figure 4 and Figure 5). Consequently, these findings suggest that the regulation of RhoA activity via changes in its binding capacity with p190RhoGAP may play an important role in prion disease.

In addition, the Rho GTPase family members Rac1 and Cdc42 have been shown to play critical roles in neuronal differentiation and development and act synergistically in the correct formation of myelin sheaths in the CNS [48]. Rac1/Cdc42 and RhoA antagonistically regulate neuronal differentiation, and their antagonistic regulation is essential for neurite outgrowth. RhoA inactivation coupled with Rac1 or Cdc42 activation enhances neurite outgrowth by the formation of point contacts and stabilization of membrane protrusions [49], which can be a possible mechanism of neuronal loss in prion pathogenesis. In this study, our data demonstrated that scrapie infection induced Rac1 and Cdc42 inactivation in both scrapie-infected hippocampal neuronal cells and the brains of scrapie-infected mice (Figure S3), indicating that the modulation of Rac1 and Cdc42 activity by scrapie infection is also related to RhoA activation.

Based on previous reports, PrP^C^ expression is involved in the early phase of neuritogenesis, which is mainly maintained by the downstream activity of the RhoA/ROCK signaling pathway and the dynamics of actin microfilaments [7]. Moreover, PrP^C^ deficiency leads to RhoA/ROCK activation, which reduces actin fiber turnover and disrupts neurite outgrowth. In addition, scrapie infection-induced RhoA/ROCK activation results in a change in neuronal polarity through the loss of neuronal synapses and neuronal functions [6]. However, inhibition of RhoA and ROCK by treatments with Tat-C3 and Y27632 reduced F-actin formation and dynamics in scrapie-infected neural stem cells [6,17,50], suggesting that scrapie infection exerts its influence on cytoskeletal rearrangement through the modulation of RhoA and ROCK activity. The pharmacological inhibition of ROCK also protects against neuronal cell death, reduces the accumulation of PrP^Sc^ and prolongs survival in scrapie-infected mice [6]. These data suggest that RhoA/ROCK activation contributes to PrP^Sc^ accumulation and aggravates prion disease.

One of the pathologies in neurodegenerative diseases involves astrocyte activation, which changes the phenotype of cells and the expression of various transporters, ion channels, and receptors, playing a vital role in neuronal cell death. Excessive release of neurotransmitters by the cells of the nervous system through gap junctions accelerates the pathophysiological process, and connexins are one of the mediators prone to inducing such neurotransmitter release through gap junctions and hemichannels [24,25,51]. In particular, the involvement of Cx43 in various neurodegenerative diseases has been reported previously, along with its contribution to neuronal cell death [52]. Furthermore, various potential roles of Cx43 include a protective role of Cx43 in oxidative stress [53] and the involvement of Cx43 in neuronal differentiation [54], neurogenesis [55] and neuronal migration [56]. In addition, an increase in Cx43 levels with amyloid plaques has been identified in both human patients and animal models of neurodegenerative diseases such as Alzheimer’s disease [57]. Moreover, in a Parkinson’s disease model, Cx43 immunoreactivity was elevated in astrocytes, glial cells and the methyl-4-phenyl-1, 2, 3, 6-tetrahydropyridine (MPTP)-lesioned striatum [30]. We also demonstrated previously that the level of Cx43 was upregulated in prion disease models, but the exact mechanism of such overexpression has not been elucidated [33]. These results clearly indicate the involvement of Cx43 in the pathophysiology of various tissue conditions.

The cytoplasmic N-terminus of connexins regulates protein trafficking, oligomerization, channel gating and hemichannel docking between cells [58,59], whereas, the cytoplasmic C-terminal tail region of connexins is also involved in multiple proteomic interactions with other proteins [60], which target Cx43 to points of cell-to-cell contact and regulate gap junctional intercellular communication. The C-terminal region of Cx43 also contains multiple phosphorylation sites that are mediated by different kinases [61,62]. The phosphorylation of Cx43 can regulate its subcellular localization, gap junction assembly and the functions of interacting partners. In addition, various Cx43-interacting proteins are responsible for the development of several human diseases, among which RhoA has been previously reported to differ between healthy and osteoarthritis samples [35]. Furthermore, in previous studies, a potent mitochondrial complex I inhibitor upregulated Cx43 expression in astrocytes of a Parkinson’s disease animal model and dopaminergic neurons [63,64]. However, inhibition of RhoA using C3 inhibited Cx43 upregulation by mitochondrial complex inhibitors [63], suggesting that RhoA activity affects Cx43 expression and hemichannel function in neurodegenerative diseases. Interestingly, our study also found that the interaction between RhoA and Cx43 was increased in models of prion disease (Figure 4 and Figure 5). In addition, inhibition of RhoA and ROCK significantly decreased the RhoA/Cx43 interaction, PrP^Sc^ accumulation and Cx43 expression (Figure 6). These data indicate that changes in RhoA/ROCK activity and the RhoA-Cx43 interaction are implicated in prion disease. However, further studies will be necessary to fully understand the molecular mechanism of these interactions under physiological conditions in both healthy and diseased states.

In conclusion, our results demonstrate that RhoA/ROCK activation, which enhances Cx43 hemichannel function, is involved in the pathogenesis of prion disease, leading to neuronal cell death. These findings are important for understanding the novel mechanisms by which the RhoA/ROCK-Cx43 signaling pathway regulates prion pathogenesis and the associated signaling pathways in both in vitro and in vivo models of prion disease.

## 4. Materials and Methods

### 4.1. Materials

Y27632, Lucifer yellow and anti-Cx43 antibodies were purchased from Sigma-Aldrich (St. Louis, MO, USA). Anti-RhoA, anti-Rac1, anti-Cdc42, anti-RhoGDI, and anti-cofilin antibodies were obtained from Santa Cruz Biotechnology (Santa Cruz, CA, USA). The anti-p190RhoGAP antibody was purchased from Millipore (Lake Placid, NY, USA). Anti-phospho-RhoA (Ser188), anti-phospho-LIMK1/2 (Thr508 and Thr505), anti-LIMK1, and anti-Cx43 antibodies were purchased from Abcam (Cambridge, MA, USA). The anti-phospho-cofilin antibody (Ser3) was obtained from Cell Signaling Technology (Danvers, MA, USA).

### 4.2. Cell Culture and Maintenance of Scrapie-Infected Cultured Cell Lines

ZW13-2 (wild-type PrP^C^), a mouse hippocampal neuronal cell line, was previously established [36], and cells were grown in Dulbecco’s modified Eagle’s medium (DMEM) (HyClone, Logan, UT, USA) supplemented with 10% heat-inactivated fetal bovine serum (FBS; HyClone), 100 units/mL penicillin and 100 µg/mL streptomycin (Thermo Fisher Scientific, Rockford, IL, USA) at 37 °C under 5% CO_2._ The ZW13-2 cells were persistently infected with the 22L and 139A scrapie strains as previously described [33]. The infected cells were maintained in Opti-MEM (Sigma-Aldrich) with 10% fetal calf serum (HyClone) and subcultured every 3 days at a 1:2 split for the first 10 passages. The infected cells stably produced PrP^Sc^ for over 50 passages.

### 4.3. Animals

Male C57BL/6J mice (6 weeks old) were purchased from Raon Bio (Yongin, Republic of Korea) and housed in a clean facility under natural light-dark cycle conditions (12 h/12 h light/dark cycle). The original stocks of the 22L and 139A scrapie strains were kindly provided by Dr. Alan Dickinson of the Agriculture and Food Research Council and Medical Research Council Institute (Neuropathogenesis Unit, Edinburgh, UK). For scrapie infection, the mice were intracerebrally inoculated with 30 μL of 1% *w*/*v* brain homogenates of the 22L strain in phosphate-buffered saline (PBS, pH 7.4) using a stereotaxic apparatus (Stoelting, Wood Dale, IL, USA). The control mice received 30 μL of 1% *w*/*v* normal brain homogenate. The scrapie-infected and uninfected mice were sacrificed at 150 days post inoculation (dpi) or the terminal stage when the mice displayed typical clinical signs of the disease. All experiments were performed in accordance with Korean laws and with the approval of the Hallym Medical Center Institutional Animal Care and Use Committee (HMC2015-0-0411-3, 12 May 2015).

### 4.4. Western Blot Analysis

Cells were washed with ice-cold PBS and lysed with a modified RIPA buffer containing 50 mM Tris-HCl pH 7.4, 150 mM NaCl, 1% Triton X-100, 0.1% sodium dodecyl sulfate (SDS), 0.5% sodium deoxycholate, 1 mM ethylenediaminetetraacetic acid (EDTA), protease inhibitors (Pierce Biotechnology, Rockford, IL, USA), 1 mM Na_3_VO_4_, and 1 mM NaF. Cell lysates were centrifuged at 15,000× *g* for 15 min at 4 °C, and the protein concentrations in the supernatants were analyzed using a BCA Protein Assay Kit (Thermo Fisher Scientific). Equal amounts of proteins (40 µg/lane) were separated using sodium dodecyl sulfate-polyacrylamide electrophoresis, transferred onto 0.45-µm pore polyvinylidene fluoride (PVDF) membranes (Merck Millipore, Lake Placid, NY, USA) and blocked with 5% skim milk in 1 × PBS containing 0.1% Tween 20 (PBST) for 1 h at room temperature (RT). The following primary antibodies were added to the membranes, which were incubated overnight at 4 °C: anti-PrP (3F10) antibody [65], anti-RhoA, anti-cofilin (Santa Cruz Biotechnology), anti-phospho-RhoA, anti-phospho-LIMK1/2, anti-LIMK1, anti-GAPDH (Abcam), anti-p190RhoGAP (Merck Millipore), anti-phospho-cofilin (Cell Signaling Technology), and anti-Cx43 (Sigma-Aldrich). The membranes were washed with PBST 3 times for 10 min each and then incubated with the following secondary antibodies for 1 h: goat anti-mouse IgG or goat anti-rabbit IgG (Thermo Fisher Scientific) conjugated with horseradish peroxidase (HRP). To detect PrP^Sc^, Equal amounts of protein (40 μg of total protein) from cell lysates or brain homogenates were treated with PK (20 μg/mL) for 30 min at 37 °C. The membranes were then washed with PBST 3 times for 10 min each, and the immunoreactive bands were visualized on digital images captured with an ImageQuantTM LAS4000 imager (GE Healthcare Life Sciences, Piscataway, NJ, USA) using the EzwestLumi plus Western blot detection reagent (ATTO corporation, Tokyo, Japan). The band intensities were quantified using the ImageJ (NIH) program. Statistical analyses were performed using GraphPad Prism 4 (San Diego, CA, USA).

### 4.5. Coimmunoprecipitation

For immunoprecipitation experiments, cells and brain tissues were lysed with modified RIPA buffer containing 50 mM Tris-HCl (pH 7.4), 150 mM NaCl, 1% NP-40, 0.25% sodium deoxycholate, 1 mM EDTA, and protease inhibitors (Pierce Biotechnology). Total lysates from the cultured cells and brains were centrifuged at 15,000× *g* for 15 min, and then the supernatants were precleared with protein A-conjugated Sepharose 4B beads and normal IgG for 2 h at 4 °C. The supernatants were then incubated with new beads and the appropriate primary antibodies for 2 h at 4 °C. After incubation, the supernatants were centrifuged at 3000× *g* for 15 s, and the beads were washed with lysis buffer 3 times for 10 min. The beads were boiled with 2× Laemmli sample buffer containing 2-mercaptoethanol for 15 min at 95 °C. The samples were electrophoresed and then analyzed by Western blot with the appropriate antibodies.

### 4.6. Glutathione-s-Transferase (GST) Pull-Down Assay for Detecting RhoA, Rac1, and Cdc42 Activity

Cells were harvested and washed with PBS, lysed in binding/washing/lysis buffer containing 25 mM Tris-HCl (pH 7.4), 150 mM NaCl, 5 mM MgCl_2_, 1% NP-40, 1 mM DTT, 5% glycerol, 1 mM EDTA, 1 mM ethylene glycol-bis (2-aminoethyl ether)-*N,N,N′,N′*-tetraacetic acid with protease inhibitors (Pierce Biotechnology), 10 mM NaF, and 1 mM Na_3_VO_4,_ and centrifuged at 13,000× *g* for 10 min at 4 °C. The supernatant was collected and incubated with GST-rhotekin-RBD to detect RhoA-GTP or GST-p21-activated kinase1 (PAK1)-PBD to detect Rac1-GTP and Cdc42-GTP. The beads were washed with binding/washing/lysis buffer 3 times. The bound proteins were eluted with 2× Laemmli sample buffer by boiling, and the samples were electrophoresed and analyzed by Western blotting with the appropriate antibodies.

### 4.7. Immunofluorescence Staining

ZW13-2 cells (5 × 10^4^ cells/35-mm dish) were rinsed with 1× PBS 3 times for 10 min each and then fixed with 4% paraformaldehyde (PFA) for 10 min. The fixed cells were rinsed with 1× PBS 3 times for 10 min each and then permeabilized with 1× PBS containing 0.1% Triton-X 100 for 10 min at RT, and then cells were blocked with 1× PBS containing 1% BSA and 5% goat serum for 1 h at RT after being rinsed with 1× PBS 3 times for 10 min each. After the cells were blocked, they were incubated with the following primary antibodies in 1% BSA and 5% goat serum in 1× PBS overnight at 4 °C: anti-Cx43 (Abcam) and anti-RhoA (Santa Cruz). Cells were subsequently incubated with either Alexa Fluor 488 goat anti-mouse IgG antibodies (Invitrogen) or Alexa Fluor 568 goat anti-rabbit IgG for 1 h and then incubated with Alexa 488 and Alexa 568 fluorescent secondary antibodies (Thermo Fisher Scientific) for 1 h. Control reactions omitting the primary antibodies resulted in no labeling with the secondary antibodies (data not shown). After being rinsed with PBS, cells were mounted in 4′,6-diamidino-2-phenylindole (DAPI)-containing Vectashield mounting medium (Vector Laboratories, Burlingame, CA, USA) to label the nuclei and visualized using an LSM700 confocal laser scanning microscope (Carl Zeiss, Oberkochen, Germany).

### 4.8. In Situ Proximity Ligation Assay

The proximity of RhoA and Cx43 proteins was examined using a Duolink in situ proximity ligation assay (PLA) kit (Sigma-Aldrich) according to the manufacturer’s instructions. Briefly, control or scrapie-infected cells were fixed with 4% PFA for 10 min and then permeabilized with 1× PBS containing 0.1% Triton-X 100 for 10 min at RT. Cells were sequentially incubated with a rabbit anti-RhoA antibody (1:200) and a monoclonal mouse anti-Cx43 antibody (1:200) overnight at 4 °C. Species-specific secondary antibodies linked to specific oligonucleotides (PLA probes) were added, and the cells were incubated for an additional 60 min at 37 °C. Ligation of PLA probes was performed by the addition of a solution containing ligase to tissue sections at 37 °C for 30 min. Finally, signal amplification was accomplished by rolling circle amplification of ligated PLA probes at 37 °C for 90 min. Sections were stained with DAPI for cell nucleus visualization. The addition of isotype control immunoglobulins instead of primary antibodies was used as a negative control. Cells were imaged using an LSM700 confocal laser scanning microscope. For each sample, five different areas were examined. The PLA dots/cells in each panel were measured and quantified for each group using the ImageJ (NIH) program. Statistical analyses were performed using GraphPad Prism 4 (GraphPad software, La Jolla, CA, USA).

### 4.9. Dye-Uptake Assay

To evaluate Cx43 hemichannel function, an uptake assay using the hemichannel-permeable reporter dye ethidium bromide (EtBr) was performed as previously described [33]. Cells were seeded onto coverslips at 2 × 10^4^ cells per well in 24-well plates, incubated in the presence or absence of a RhoA-specific inhibitor (Tat-C3, 1 µg/mL) and a Rho-associated kinase inhibitor (Y27632, 10 μΜ) for 6 h and then incubated with 5 μM EtBr for 5 min at 37 °C. Cells were washed with Hank’s balanced salt solution (HBSS), fixed with 4% paraformaldehyde in PBS for 15 min at RT and washed with HBSS. The dye-uptake assay was performed using an LSM700 confocal laser scanning microscope. The mean fluorescence intensity was quantified in arbitrary units in 65,000 shades of gray with ImageJ program. Statistical analyses were performed using GraphPad Prism 4.

### 4.10. Scrape-Loading Dye Transfer Assay

To determine Cx43 hemichannel function, an uptake assay using the gap junction permeable reporter dye Lucifer yellow (LY) (Sigma-Aldrich) was performed as previously described [43]. Cells were pretreated with RhoA/ROCK inhibitor for 6 h and then incubated with 0.01% LY in PBS for 5 min at 37 °C. Cells were washed with HBSS, fixed with 4% PFA for 15 min and observed under an LSM700 confocal laser scanning microscope. The distances of LY diffusion after scrape loading were measured in at least twenty random areas from each sample, and the fluorescence intensity was assessed using INFINITY ANALYSIS software (Lumenera, Ottawa, ON, Canada) and compared between the control and infected cells with or without each treatment.

### 4.11. Statistical Analysis

Statistical analyses were performed, and graphs were generated using GraphPad Prism software (GraphPad Prism 4, GraphPad software, La Jolla, CA, USA). Statistical differences were determined by one-way analysis of variance (ANOVA) followed by Tukey’s post hoc test. The data are presented as the means ± SEM. Statistical significance was reached at *p* < 0.05.

## Supplementary Materials

Supplementary materials can be found at https://www.mdpi.com/1422-0067/21/4/1255/s1.

## Abbreviations

GAP	GTPase-activating protein
ROCK	Rho-associated kinase
RhoGDI	Rho GDP-dissociation inhibitor
GST-Rhotekin-RBD	Glutathione S-transferase-Rhotekin-Rho-binding domain
GST-Rhotekin-PBD	Glutathione S-transferase-p21 binding domain
GJA1	Gap junction alpha-1 protein
PDK1	ROCK-3-phosphoinositide-dependent kinase 1
