# Supplementary material for: RhoA/ROCK Regulates Prion Pathogenesis by Controlling Connexin 43 Activity
Source: Int J Mol Sci. 2020 Feb 13;21(4):1255. doi: 10.3390/ijms21041255 (PMC7072953; doi:10.3390/ijms21041255)
Supplement: Supplementary file 1 [file ijms-21-01255-s001.pdf]

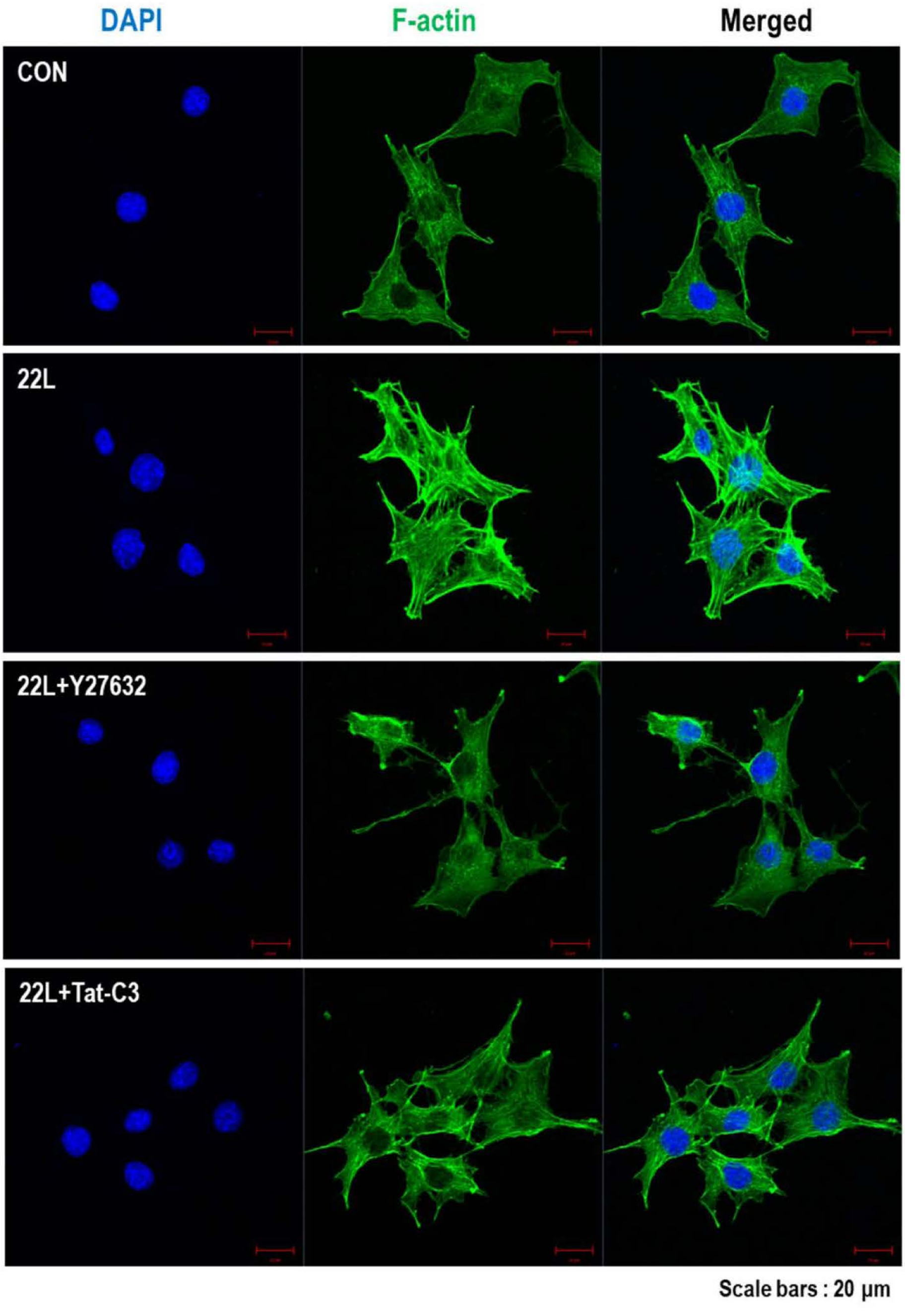

Figure S1. Inhibition of RhoA and ROCK affects the F-actin formation in scrapie-infected hippocampal neuronal cells. Control (CON) and 22L scrapie-infected ZW13-2 hippocampal neuronal cells were incubated with or without 10 μM Y27632 or 1 μg/ml Tat-C3 for 24 h and analyzed F-actin formation by immunocytochemical staining. Cells were fixed with 4% PFA and permeabilized with 0.2% Triton X-100 in PBS. F-actin was stained with Alexa Fluor 488-phalloidin and DAPI was used to counterstain the nuclei. All pictures are representative of multiple images from three independent experiments (scale bars, 20 μm).

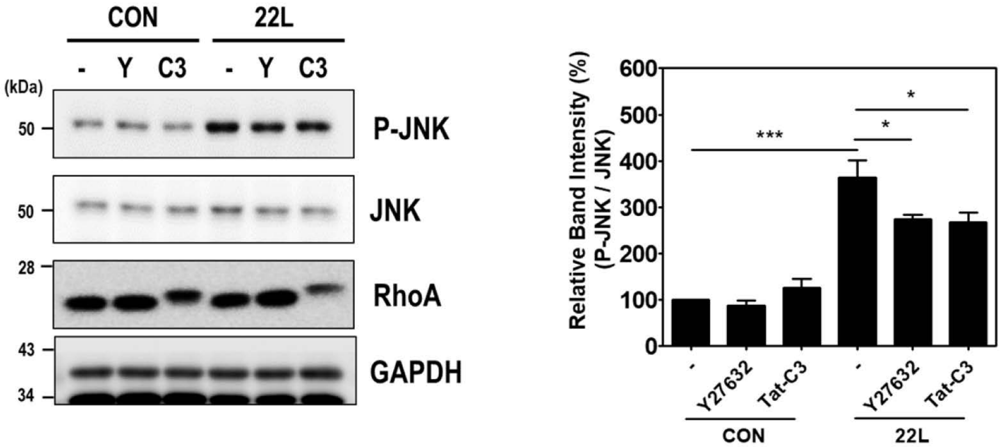

Figure S2. *Inhibition of RhoA and ROCK affects the JNK phosphorylation in scrapie-infected hippocampal neuronal cells.* Control (CON) and 22L scrapie-infected ZW13-2 cells were incubated with or without 10 μM Y27632 (Y) and 1 μg/ml Tat-C3 (C3) for 24 h. Phosphorylation levels of JNK was analyzed by Western blot. GAPDH was used as a loading control. The intensities of the bands in each panel were measured and quantified. The values were expressed as the mean ± SEM of three independent experiments. Statistical data were obtained by one-way ANOVA test with Tukey's *post hoc* test (n=3, *$p < 0.05$; ***$p < 0.001$).

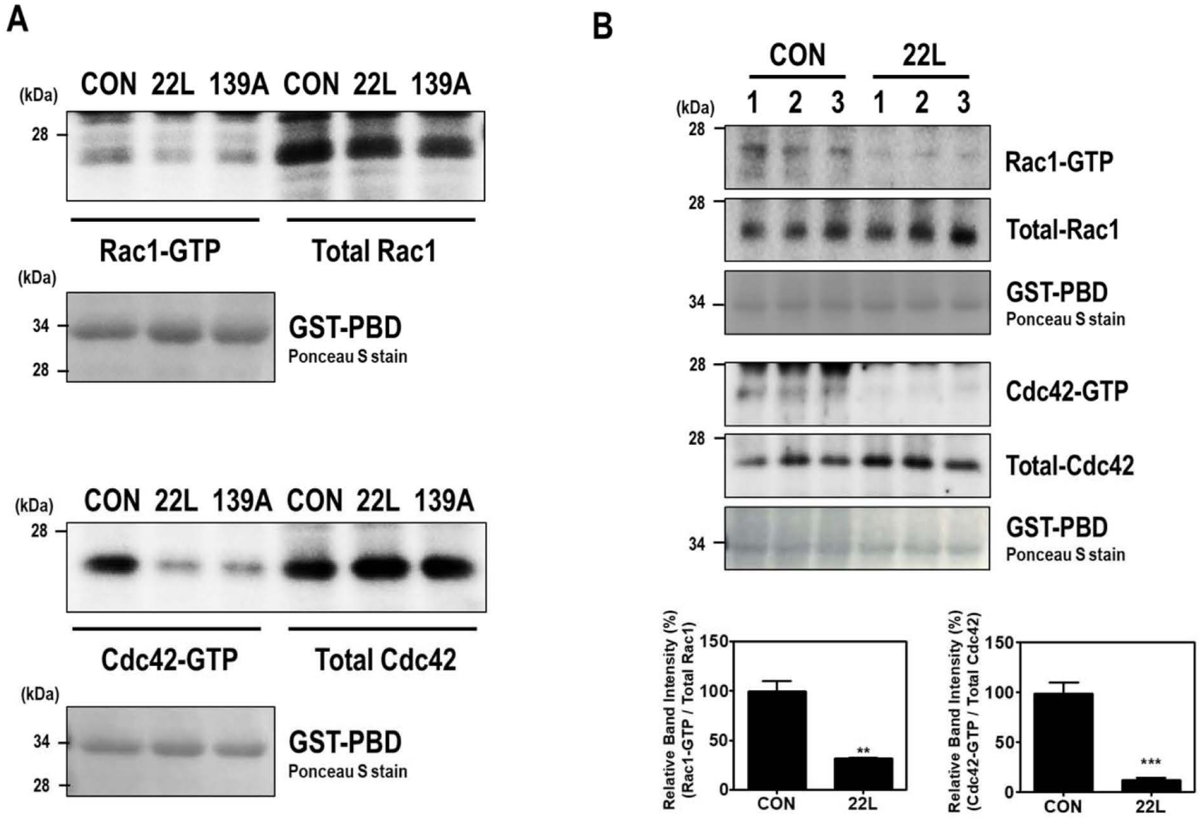

Figure S3. *Scrapie infection is involved in Rac1 and Cdc42 activation.* (A and B) Detection of Rac1-GTP and Cdc42-GTP by GST-p21-activated kinase 1 (PAK1)-PBD pull-down assay in ZW13-2 cells with or without scrapie infection (22L or 139A) (A) and the brains of 22L scrapie-infected mice (B). The data are expressed as the mean ± SEM of three independent experiments. Statistical differences were determined by one-way ANOVA test with Tukey's *post hoc* test (n=3, ** $p < 0.01$; *** $p < 0.001$).
